# An Investigation into Mechanical Properties and Printability of Potential Substrates for Inkjet Printing of Orodispersible Films

**DOI:** 10.3390/pharmaceutics13040468

**Published:** 2021-03-30

**Authors:** Erna Turković, Ivana Vasiljević, Milica Drašković, Nataša Obradović, Dragana Vasiljević, Jelena Parojčić

**Affiliations:** 1 Department of Pharmaceutical Technology and Cosmetology, Faculty of Pharmacy, University of Belgrade, Vojvode Stepe 450, 11221 Belgrade, Serbia; ivana.vasiljevic@pharmacy.bg.ac.rs (I.V.); milica.draskovic@pharmacy.bg.ac.rs (M.D.); dragana.vasiljevic@pharmacy.bg.ac.rs (D.V.); jelena.parojcic@pharmacy.bg.ac.rs (J.P.); 2 Department of Chemical Engineering, Faculty of Technology and Metallurgy, University of Belgrade, Karnegijeva 4, 11000 Belgrade, Serbia; ntomovic@tmf.bg.ac.rs

**Keywords:** inkjet printing, printing substrates, mechanical properties, orodispersible films, structured orodispersible film templates, wafer edible sheets

## Abstract

Inkjet printing is novel approach in drug manufacturing that enables dispensing precise volumes of ink onto substrates. Optimal substrate properties including suitable mechanical characteristic are recognized as crucial to achieve desired dosage form performance upon administration. Identification of relevant quality attributes and their quantification is subject of intensive scientific research. The aim of this work was to explore applicability of different materials as printing substrates and explore contribution of the investigated substrate properties to its printability. Substrates were characterized with regards to uniformity, porosity, disintegration time, mechanical properties and drug dissolution. Experimentally obtained values were mathematically transformed and the obtained results were presented as relevant radar charts. It was shown that structurally different substrates may be employed for orodispersible films inkjet printing. Main disadvantage of single-polymer films was low drug load, and their printability was dependent on film flexibility and mechanical strength. Structured orodispersible film templates exhibited favorable mechanical properties and drug load capacity. Wafer edible sheets were characterized with high mechanical resistance and brittleness which somewhat diminished printability, but did not hinder high drug load. Obtained results provide insight into application of different materials as printing substrates and contribute to understanding of substrate properties which can affect printability.

## 1. Introduction

Inkjet printing (IJP) is a commonly used digital fabrication technique which allows processing and precise deposition of various liquid materials onto suitable substrates. It is recognized as a novel promising technology for medicine manufacture providing patient-centric drug delivery, and individualization of therapy through flexible dosing of multiple, usually high potency active pharmaceutical ingredients (API) in accordance with the patient specific needs [1,2]. The principle of IJP is that the ink, which contains active pharmaceutical ingredient/s, is precisely transferred onto the selected substrate. In order to obtain targeted drug product profile, both ink formulation, as well as substrate properties should be carefully considered [3,4,5].

Iftimi et al. defined the ideal printing substrate as a uniform, edible and flexible porous open-pore carrier that could be produced in large sheets [6]. They qualitatively summarized specific substrate properties that are needed in order to obtain optimal printing substrate. The importance of substrate mechanical stability was highlighted as it ensures printing of high volumes of the API-containing ink. Morphology, water penetration rate, low hygroscopicity, porosity, swelling index and fast dissolution were also evaluated as factors that can affect substrate printability [6]. Mechanically stable substrates enable film printing, distribution and administration without final dosage form damaging, i.e., they should be flexible enough, but still resistant enough to ensure maintenance of dosage form integrity during printing and handling steps [7]. Methodology for printing substrate mechanical properties evaluation and relevant specifications are subject of intensive investigation. Visser et al. [8] quantified mechanical properties of polymer films and reported that tensile strength higher than 2 MPa, elongation at break higher than 10% and Young’s modulus lower than 430 MPa can be considered as optimal for drug-free films handling, however, these boundaries should be further evaluated with respect to their applicability for characterization of polymer films intended for use as printing substrates.

Apart from the mechanical strength which reflects the substrate flexibility/rigidity, it is recognized that the important characteristic for effective printing onto the thin films is porous structure which facilitates penetration of the API-containing ink [9]. Furthermore, printing substrates should be relatively thick as opposed to conventional oral strips to enable printing of higher drug doses, and prevent disintegration upon contact with the ink [6]. Thus, the main challenge for orodispersible films printing substrates is to prevent disintegration, rupturing, tearing or winding during printing, while maintaining rapid disintegration required for orodispersible dosage forms [10].

The most common substrates used for IJP are orodispersible thin films prepared using different film-forming polymers. It was shown that API printing onto placebo orodispersible thin films, may overcome certain limitations related to film casting in terms of product thickness and content uniformity, the associated dose variation, and unacceptable material waste [9,11]. It was noted that orodispersible thin films need to be improved in order to further increase the amount of API absorbed and prevent ink leakage through backside of the printing substrate [7]. Enhanced film porosity was associated with better control of ink deposition and the ability to entrap higher amount of inkjet-printed API inside the matrix, although mechanical properties might be somewhat diminished [10].

Structured orodispersible film templates (SOFTs) have been recently introduced as highly porous substrates which enable increased drug load without compromising its mechanical properties. Steiner et al. [12] prepared SOFTs by casting dispersion of hydroxypropyl methyl cellulose in hydroxypropyl cellulose ethanolic solution in order to form a rougher film surface with open pore structure on the top side enabling API-containing ink to be filled into the pores, and the closed bottom side to circumvent leakage. Open pore structure is crucial for ink penetration, as closed pore structure with a continuous film on its surfaces restricts ink penetration [13].

Apart from the casted substrates such as orodispersible thin films or SOFTs, commercially available edible papers, which are often used in the food industry to decorate baked goods and other food products, might be, also, used as printing substrates. Wafer edible sheets and rice papers have been previously used as printing substrates for API-containing inks due to their porous structure and the ability to absorb relatively high amounts of liquid [14,15,16,17].

The aim of this study was to explore applicability of different orodispersible thin films, structured orodispersible film templates and wafer edible sheets as printing substrates for IJP. Additionally, printability of substrates was evaluated with respect to porosity, thickness, drug load capacity and the ability of orodispersible thin films to withstand mechanical stress and deformation when passing through printer rollers, expressed as relevant mechanical properties, including film tensile strength, Young’s modulus, elongation at break and complex modulus.

## 2. Materials and Methods

### 2.1. Materials

Four hydrophilic polymers were investigated as single film-forming agents or polymer blends for printing substrates preparation: (1) hydroxypropyl cellulose (HPC, Klucel^®^ GF, Ashland™, Wilmington, DE, USA), (2) polyethylene glycol–polyvinyl alcohol graft copolymer (PVA-PEG, Kollicoat^®^ IR, BASF, Ludwigshafen, Germany), (3) maltodextrin (MDX, Glucidex IT6, Roquette, Lestrem, France) and (4) sodium alginate (SA, Fisher Scientific, Waltham, MA, USA), as well as three commercially available wafer edible sheets (Easy Bake, UK, Edible print supplies, Birstall, The United Kingdom). Ink formulation contained caffeine anhydrous (CAF, Sigma-Aldrich Chemie GmbH, Munich, Germany) as the selected model drug, dissolved in the 7:3 mixture of ethanol (≥99.8%, Honeywell, Charlotte, NC, USA) and glycerol, 85% (*w/w*) (Ph. Eur.).

Simulated salivary fluid pH 6.75 [18] prepared with sodium chloride, potassium phosphate monobasic, disodium hydrogen phosphate, hydrochloric acid (Sigma-Aldrich Chemie GmbH, Munich, Germany) and purified water (Ph. Eur.) was used as drug release media.

### 2.2. Methods

#### 2.2.1. Printing Substrate Preparation

Single-polymer casting dispersions were prepared by dispersing relevant polymer in water heated to 50 °C (in the case of PVA-PEG, SA and MDX) or 70 °C (followed by rapid cooling, in the case of HPC). Dispersions were stirred on the magnetic stirrer (IKA RCT standard, Staufen, Germany) until homogenization.

Polymer blend casting dispersions were prepared by dispersing PVA-PEG, SA or MDX in HPC ethanolic solution followed by continuous stirring on the magnetic stirrer for one hour.

Prepared dispersions were casted on a unit-dose plexiglas plates as described by Drašković et al. [19]. The films were left to dry under ambient conditions during 24 h, cut into pieces of defined size (2.5 by 2.5 cm), packed and stored in a desiccator. Commercial wafer edible sheets were manually cut into 2.5 by 2.5 cm individual films.

#### 2.2.2. Ink Formulation Preparation

Based on the preliminary studies (data not shown), ethanol:glycerol mixture (7:3) has been selected as liquid vehicle for inkjet printing. Then, 10 mg/mL of CAF was dissolved in the solvent mixture. Hydrosoluble food dye containing water, propylene glycol, E 124 and E 122 (Aroma, Krusevac, Serbia) was added in order to facilitate visualization of the printed patterns. Further details on ink characterization are presented in the Table S1 and Figure S1 from the Supplementary Material.

#### 2.2.3. Drug Printing

Thermal inkjet printer Canon^®^ IP 1300 (Canon, Tokyo, Japan) was used. Cartridges were adapted by cutting the top cap, removing the ink sponges and pads and rinsing the empty cartridges with absolute ethanol and purified water. Rectangular printing pattern (2.5 by 2.5 cm) was designed in Microsoft^®^ Office Word 2019 (Microsoft Inc., Albuquerque, NM, USA). In the preliminary study (data not shown), best print quality was obtained using black cartridge (BC-3e BK) solely, therefore, the selected pattern was painted black. Printer settings were adjusted to the following option: High print quality/glossy photo paper/grayscale printing. Printing process consisted of five printing cycles, with 15 min drying step between each cycle. Plain paper was used as a support for individually casted substrates in order to accomplish precise printing. Paper was preprinted with designed pattern and substrates were attached onto paper with an adhesive tape.

#### 2.2.4. Printing Substrate Characterization

##### Uniformity

Film uniformity was assessed based on the individual films weight, thickness and printed pattern appearance. Thickness was measured at five positions (four corners and one central point) using micrometer screw Insize 3203-25 A (Insize, Suzhou, China). Film weight and thickness are presented as mean values (±SD) of ten replicate measurements. Printed pattern appearance was assessed by visual inspection of the uniformity of color and edges definition, after five printing cycles. Uniformly distributed color without smearing was considered as acceptable appearance (marked with “+”), while visible splashes of color indicated poor appearance (which was denoted as “−”). The same marking system was used to denote printed patterns edges definition.

##### Porosity

Porosity was determined as a relative weight difference of the investigated samples following 24 h immersion in the paraffin oil as described by Khorasani et al. [20]. Measurements were performed in triplicate and presented as the mean value (±SD).

Additionally, porosity was investigated using the ImageJ software package 1.51k (National Institutes of Health, Stapleton, NY, USA). Micrographs obtained by trinocular microscope (SZM-168-TL, Motic, Barcelona, Spain) were converted to 8-bit images and the threshold was adjusted to color empty space within the structure, while solid parts remained black. The software was used to calculate fraction of pores in the investigated sample. Relationship between the experimentally determined porosity values and those estimated by image analysis was explored using linear regression analysis.

##### Image Analysis

Trinocular microscope (SZM-168-TL, Motic, Barcelona, Spain) and scanning electron microscope-SEM (JEOL, JSM-6390 LV, Akishima, Japan) were used to visualize the drug-free samples surface morphology. SEM sample preparation included cutting samples into small pieces and fixing them to the sample holder with double-adhesive carbon tape. After that, samples were coated with gold alloy on sputter coater (Baltec SCD 005, Baltec, Buffalo Grove, IL, USA) to improve their conductivity during recording. Smile Shot^TM^ software was used for obtaining images.

Polarized microscopy (Olympus BX51-P polarized microscope, Olympus, Tokyo, Japan) was employed to detect presence of CAF crystals in the printed samples. For polarized light microscopy a Sony DXC-950P digital camera (Sony, Tokyo, Japan) was used with CellSens Entry 3.1 software (Olympus, Tokyo, Japan).

##### Disintegration

Disintegration time (DT) of the investigated samples was recorded before and after five printing cycles. Disintegration test for orodispersible dosage forms developed by Preis et al. [21] was employed using 500 mL of the simulated salivary fluid heated to 37 ± 0.5 °C in the compendia disintegration apparatus (Erweka ZT52, Langen, Germany). Individual films were fixed with a holder attached to the upper part and the magnet (3 g) attached to its bottom side. Disintegration endpoint was determined as the time when the magnet attached to the investigated sample dropped down. Six samples were tested, and the results are reported as mean value (±SD). Paired t-test was used for comparison of disintegration time values before and after printing.

##### Mechanical Properties

Mechanical properties of the investigated printing substrates were evaluated using the Precision universal tester (Shimadzu AG-X plus, Shimadzu Corporation, Kyoto, Japan). The test was performed according to the ISO 527-3 regulation [22]. Samples were cut in the bone shaped specimens, clamped with the film extension grip which moved at a speed of 1 mm/min until sample breakage. The measurements were performed in triplicate. Sample tensile strength (TS), elongation at break (EB) and Young’s modulus (YM) were calculated according to the Equations (1)–(3).

TS (MPa) = F/A(1)

where F is maximal applied load and A cross-sectional area.

EB (%) = 100 × (∆L_0_)/L_0_ (2)

where ∆L_0_ is the extension and L_0_ is the original length

YM = (σ_2_ − σ_1_)/(ε_2_ − ε_1_) (3)

where σ_2_ − σ_1_ represents the applied stress over strain ε_1_ and ε_2_.

Viscoelasticity of the investigated samples was evaluated based on the complex modulus (G*) values determined by oscillatory rheometry (RheometerRheolab MC 120, PaarPhysica, Stuttgart, Germany) using the parallel plate measuring system MP50 (diameter 12.5 mm, gap 50 μm) with samples placed into frames to prevent drifting.

Oscillatory measurements were performed to determine linear viscoelastic region of the investigated samples (amplitude sweep). After linear viscoelastic region was determined and all the measurements were performed at the constant strain (1%) within frequency range 0.1–10.0 Hz to estimate the impact on the change in storage (elastic) modulus (G′) and loss (viscous) modulus (G″) values.

G* is calculated using the following equation [23]:

|G*|=√((G′)2 + (G″)2)(4)

Measurements were performed in triplicate and the results expressed as mean value (±SD).

##### Drug Load

Drug load achieved after five printing cycles was determined by dispersing individual film in 10 mL of purified water on the laboratory shaker (KS 260 basic, IKA VR-Werke GmbH, Staufen, Germany) at 250 rpm. Obtained samples were filtered through a 0.45 mm filter (Millipore, Bedford, MA, USA), properly diluted and assayed for CAF at 273 nm using UV-spectroscopy (UV spectrophotometer EvolutionTM300, Thermo Scientific, Waltham, MA, USA). Reference measurements were done with drug-free substrates used as a blank in order to eliminate interference of substrate components. Test was performed in triplicate.

##### In Vitro Drug Dissolution

CAF dissolution from the printed samples was studied in the small volume dissolution setup consisting of 100 mL laboratory glasses immersed in the temperature-controlled shaker (LSB Aqua Pro18, Grant, Shepreth, UK) agitated at 110 rpm. Investigated samples were attached to the bottom of the glass with the printed side facing up, and 50 mL of simulated salivary fluid (pH 6.75, 37 ± 0.5 °C) was carefully added. Then, 2 mL samples were withdrawn manually at the pre-determined time intervals. CAF concentration was determined using UV spectrophotometer (EvolutionTM300, Thermo Scientific, Waltham, MA, USA) at 273 nm (drug-free substrates were used as blank). The test was performed in triplicate, and the results are expressed as the mean values (±SD).

##### Printability Evaluation

Experimentally obtained results for porosity (POR), thickness (TH), EB, TS, YM, G* and drug load (DL) were mathematically transformed onto the 0–100% scale in order to perform comparative evaluation of the investigated substrates printability. Estimated printed pattern appearance (PPA) was assigned with the value 0, 5 or 10 if the sample scored none, one or two pluses, respectively. PPA values were, also, transformed onto the 0–100% scale and value 100% was considered as favorable, as it indicated both the uniformity and precision of drug distribution. Furthermore, high porosity and substrate thickness were recognized as factors that contribute to higher drug load [6,12]. Mathematical transformation of experimentally obtained mechanical characteristics was based on the boundary values recommended by Visser et al. [8]. Accordingly, the target value for tensile strength was set to 2 MPa or higher, while in the case of elongation at break it was equal or higher than 10%. Young’s modulus was considered more satisfactory in the case of lower values, while values higher than 400 MPa were unfavorable. Complex modulus might be useful in prediction of substrates resistance to deformation. Hence, higher values are favorable, as they can indicate substrates ability to withstand repeated printing cycles [19]. Factors affecting substrate printability are represented as radar charts which provide multivariate data visualization, where larger chart area indicates better printability.

## 3. Results and Discussion

### 3.1. Printing Substrate Preparation

Ten printing substrate samples were prepared or purchased from the market. Sample subset I included four polymer films prepared using HPC, PVA-PEG, SA and MDX as single film-forming agents in the concentration of 7%, except in the case of MDX samples where, due to film sticking, polymer concentration was set to 5%. The subset II included three samples prepared as structured orodispersible film templates containing polymer blends in which HPC was used as binder with the addition of PVA-PEG, SA and/or MDX as particulate matrix material. The subset III included three wafer edible sheets purchased from the market. Composition of the investigated samples is presented in Table 1.

### 3.2. Uniformity

Weight, thickness and the estimated printed pattern appearance of the investigated samples are presented in Table 2. The subset I samples were characterized with lower thickness (ranging from 69 to 124 μm), and film weight when compared to the subset II samples which exhibited higher and more variable thickness (ranging from 309 to 481 μm) due to the presence of dispersed polymer particles on top of the HPC base which resulted in rough and uneven film surface. The commercial wafer edible sheets (sample subset III) also varied in composition and thickness, which ranged from 264 to 502 μm.

Substrate S5 exhibited high level of uniformity with regards to color deposition after five printing cycles. Occasional splashes of ink were seen on the surface of samples S1, S7 and S8. Well defined edges of the printed patterns indicate that ink was not smeared and removed by printer roller. Inconsistency in printed pattern color deposition and edges definition was observed for substrates S1 and S3, hence they were marked as “poor”.

### 3.3. Porosity

Experimentally obtained and calculated substrate porosity values are presented in Table 2. Orodispersible thin films (i.e., subset I) exhibited poor oil absorption capacity and porosity values were in the range from 0.8 to 1.8%. Porosity values estimated by image analysis were consistent with the experimentally determined results and ranged from 0.3 to 3.1%. The subset II samples exhibited diverse experimentally determined porosity values ranging from 3% (which is close to the determined values for orodispersible thin films) in the case of S6 sample, to more than 6% (which is comparable to wafer edible sheets) for the samples S5 and S7. Sample S6 contained SA polymer as a particulate matrix material characterized with good swelling and gelling properties. It can be anticipated that the reason for a more compact top layer being associated with lower porosity of the SA containing sample (S6) when compared with samples S5 and S7 containing, respectively, PVA-PEG and MDX as particulate matrix material might be polymer swelling and gelation during the film casting and drying, as discussed by Shi et al. [24].

Commercial wafer edible sheets (i.e., the subset III samples) exhibited high porosity values, ranging from 6.1 to 9.7% as estimated based on the oil absorption capacity, and 30.4 to 41.1% estimated by image analysis. However, linear regression analysis indicated high correlation between experimentally and ImageJ determined porosity (y = 4.88x – 3.44, R = 0.98).

### 3.4. Image Analysis

**Figure 1:**
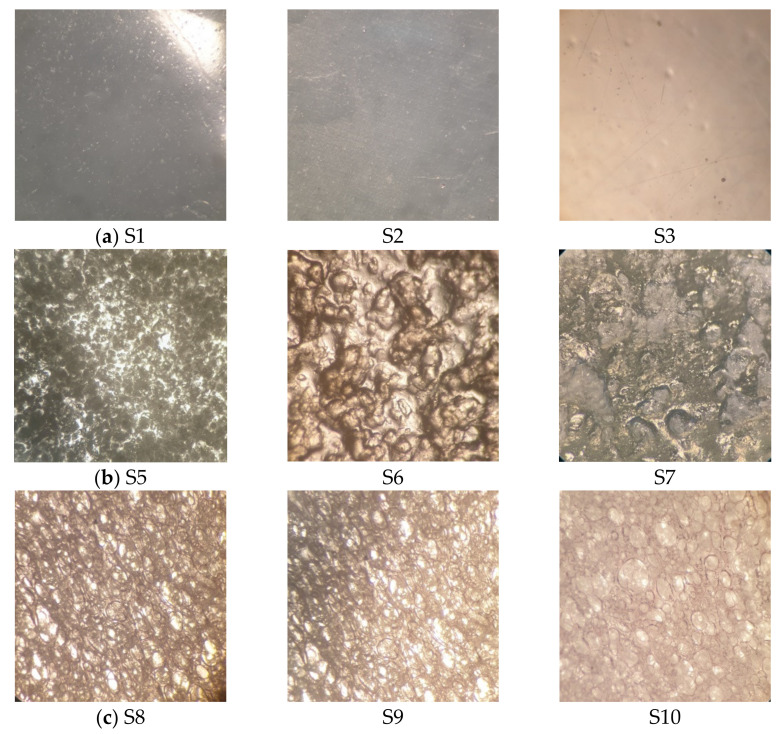
Photomicrographs (50×) of the investigated printing substrates obtained by trinocular microscope: (**a**) subset I; (**b**) subset II and (**c**) subset III (for the sample composition refer to Table 1).

Photomicrographs of the investigated samples obtained by the trinocular microscope are presented in Figure 1. While the subset I samples (Figure 1a) reflected the image of transparent material, without any inner structure, photomicrographs of the subset II and III samples (Figure 1b,c) showed notable differences with respect to their inner structure and pore distribution.

Open pores which allow light transition were visible in the samples S5 (HPC-PVA-PEG SOFT) and S7 (HPC-MDX SOFT), with more uniform pore distribution evident for the sample S5. Such structure is in accordance with relatively high porosity of these samples. Sample S6 (HPC-SA SOFT) exhibited compact inner structure without any transparent sections which is in accordance with low porosity value obtained for this sample. Commercial wafer edible sheets exhibited uniform distribution of open pores which is consistent with their high porosity values. In addition, in contrast to the subset II samples, subset III samples enabled intensive light transition, probably due to the lack of closed bottom side which is attributed to SOFTs.

**Figure 2:**
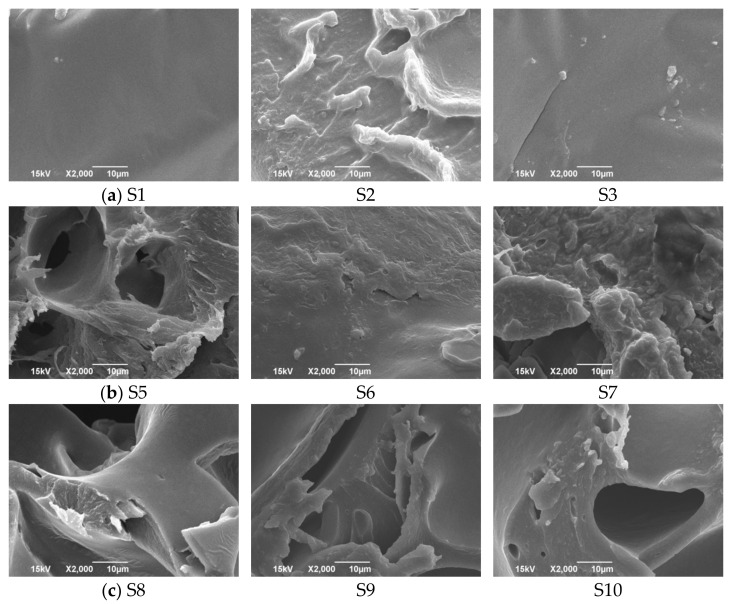
SEM Photomicrographs of the investigated substrates: (**a**) subset I; (**b**) subset II and (**c**) subset III.

The cross-sectional structures of the samples were assessed using SEM (Figure 2). Subset I samples appeared dense with no inner pores, but there were differences in microstructure. Sample S1 had completely smooth cross-sectional surface while S2 had wrinkled appearance with higher surface area. S3 had small cracks throughout cross sectional area which might indicate stiff structure. Sample S5 exhibited uniformly distributed pores and formed wrinkled structure. Sample S6 cross section appeared homogeneous and similar to orodispersible thin films, which was in agreement with the estimated porosity values. In the case of sample S7 porous inner structure was observed, but pores were irregular and sporadically distributed. Similar composition of subset III samples resulted with similar microstructure with noticeable layers. High porosity might be due to an open space between layers.

**Figure 3:**
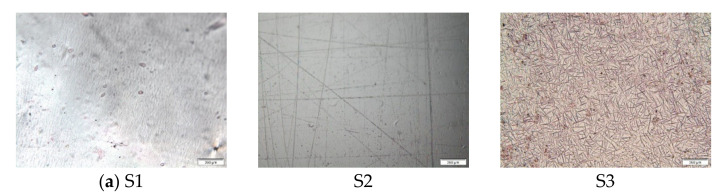

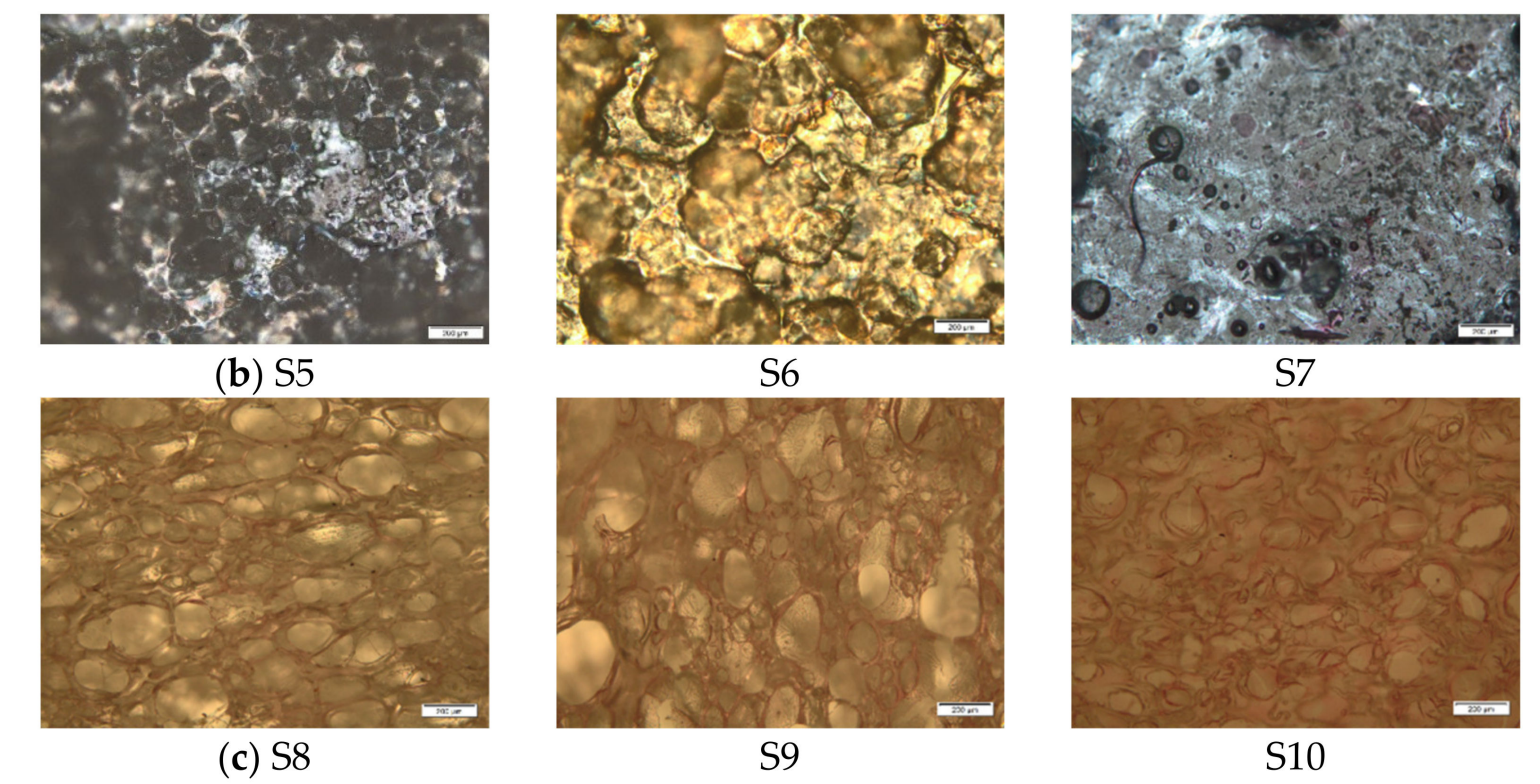
Photomicrographs of the printed samples obtained using polarized light microscopy (200 µm): (**a**) subset I; (**b**) subset II and (**c**) subset III.

Polarized light photomicrographs of the evaluated printed films are presented in Figure 3. The obtained photomicrographs provide insight into the presence of needle-shaped CAF crystals only in the sample S3 (SA) while crystals were not visible in other investigated samples. Crystallization of CAF at the surface of the S3 substrate might be associated with the poor drug adhesion onto the printing substrate and incorrect dosing. It was previously assumed that HPC polymer has the ability to inhibit drug recrystallization [10], which might explain why CAF crystals were not observed in the samples containing HPC. Additionally, it was reported that penetration of ink into the porous printing substrates is associated with altered crystallization behavior compared to printing on nonporous substrates [25]. Hence, it might be assumed that CAF entrapped into the porous substrate will not recrystallize. This is in accordance with the absence of visible CAF crystals in the subset II and III samples.

### 3.5. Disintegration

Disintegration times of the drug-free and printed samples are presented in Table 3. Sample S4, prepared from single-polymer dispersion, ruptured upon first contact with the ink and was eliminated from further evaluation. Five printing cycles had no significant influence on disintegration time of the other investigated samples (*p* = 0.75 for paired samples *t*-test). Additionally, all samples fulfilled pharmacopeial requirement for orodispersible tablets disintegration [26].

Although films prepared from polymer blends dispersion (i.e., subset II samples) were thicker than the single polymer film samples (subset I) this was not associated with prolonged disintegration. Similar disintegration was observed for S1 and S5, which might be attributed to higher porosity of the sample S5, in which the open pore side enabled rapid water penetration. Samples S6 and S7 exhibited somewhat longer disintegration time possibly due to lower porosity and, consequently, slower water penetration through the top side of the samples.

The results obtained revealed that S8 and S9 samples, which contain highly porous structure with both sides open, exhibited shortest disintegration times. On the other hand, sample S10 exhibited the longest DT. Highly porous structure with both sides open was associated with very fast disintegration, while multidimensional inner structure and higher film thickness might be the reason for somewhat different behavior of the sample S10.

### 3.6. Mechanical Properties

Mechanical properties of the evaluated drug-free samples, including tensile strength, elongation at break, Young’s modulus and complex modulus are presented in Table 4. Elongation at break is described as the capacity of film to stretch before it breaks. Therefore, if the elongation at break is high, sample structure might be considered as flexible and ductile [27]. The obtained results revealed the highest EB value (272.9%) for the sample S1 which contained hydroxypropyl cellulose as the single film forming polymer. Although some level of flexibility is required for the substrate to be able to fold when it passes through the printer, extensive flexibility might cause sample stretching leading to erroneous drug disposition [28]. On the contrary, low EB determined for samples S3 and wafer edible sheets (S8–10) indicate potential rupturing during film folding due to pronounced brittleness. Elongation at break values for samples S5 and S7 were comparable (i.e., around 11%), while the sample S6 exhibited somewhat higher flexibility. The subset II samples were characterized with flexible structure, but when compared to S1 substrate it can be assumed that addition of particulate matrix material greatly reduced film flexibility. The obtained EB values indicate that the subset II substrates can fold multiple times during printing without dose disruption. The subset III samples exhibited low EB values which could potentially lead to problems during multiple printing cycles, as their lack of flexibility limits folding without breaking.

Tensile strength is defined as the maximum load force used to break the sample. Hard and brittle substrates demonstrate very high mechanical resistance [29]. Generally, somewhat higher TS is preferred in the case of printing substrates in order to avoid tearing which could result from the constant stress induced by the printing rollers. High flexibility of the sample S1 was accompanied with lower mechanical resistance compared to the other subset I samples. Sample S3 containing sodium alginate exhibited extreme brittleness and the highest strength among all the investigated samples, which might cause certain problems during repeated printing cycles. Considering that HPC presents the base polymer in the subset II samples, all substrates exhibited more flexible and less brittle structure compared to thin orodispersible films. Commercially available, edible sheets (subset III) exhibited lower TS values, which is in accordance with the literature data reported by Vakili et al. [16].

Young’s modulus is associated with film stiffness and capacity to undergo elastic deformation under applied stress [29]. Strong positive correlation between YM and TS values was established (R^2^ = 0.98), indicating that more mechanically resistant substrates are also stiffer. YM, also, represents parameter that can serve as reliable indicator of substrate durability during printing and further handling. The obtained results revealed great diversity in YM values in the subset I (i.e., single polymer films) confirming pronounced impact of polymer characteristics. Sample S1 prepared with HPC exhibited the lowest (2.99 MPa) while sample S3 prepared with SA exhibited the highest determined YM value (3498 MPa), which was in accordance with the other investigated mechanical properties. As it was previously reported, presence of MDX in the sample S7 might be related to the increased film hardness and stiffness, without affecting film flexibility as discussed by Cilurzo et al. [30]. According to the presented results, all the investigated samples, except sample S3, had YM in line with recommendation, i.e., lower than 430 MPa [8].

Complex modulus is defined as a measure of total resistance of the system to strain. It is noted that systems that have increased fraction of the dispersed phase are characterized with higher G* values, as a result of the particle–particle interaction and more rigid structure [19,31]. Low values of G*, which were determined for samples S1 and S2 indicate greater flexibility, while the highest complex modulus value observed for S6 might be related to stiffer and less flexible structure. Although Young’s modulus values of the subset III samples were comparable, substrate S10 had very high G*, probably due to higher thickness and more complex inner structure (as presented in Figure 2).

### 3.7. Drug Load

Determined drug load of the investigated orodispersible films, after five repeated printing cycles, are presented in Table 2. The obtained results revealed great inconsistency of CAF load in the investigated samples, ranging from 54.2 to 437.1 µg. In the subset I samples, lower amounts of drug were incorporated indicating that thin orodispersible films could hold ink only on the top of the surface. The only exception was evident in the sample S3 (prepared with SA), where CAF recrystallization caused facilitated removal of printed drug during successive printing cycles. Within the subset II, the highest drug content was incorporated in the sample S5 (437.1 µg), which is in accordance with the highest porosity and uniform distribution of open pores observed (Figure 2a). Commercially available edible substrates (subset III) are produced with intention to reach high sorption capacity for edible inks. It was noticed that level of porosity affects the amount of drug incorporated. According to the presented results it might be assumed that substrate porosity is the main indicator of the drug load capacity, so the sample S5 with uniform and high porosity and the highest drug content incorporated can be considered as favorable among the presented samples.

### 3.8. In Vitro Drug Dissolution

**Figure 4:**
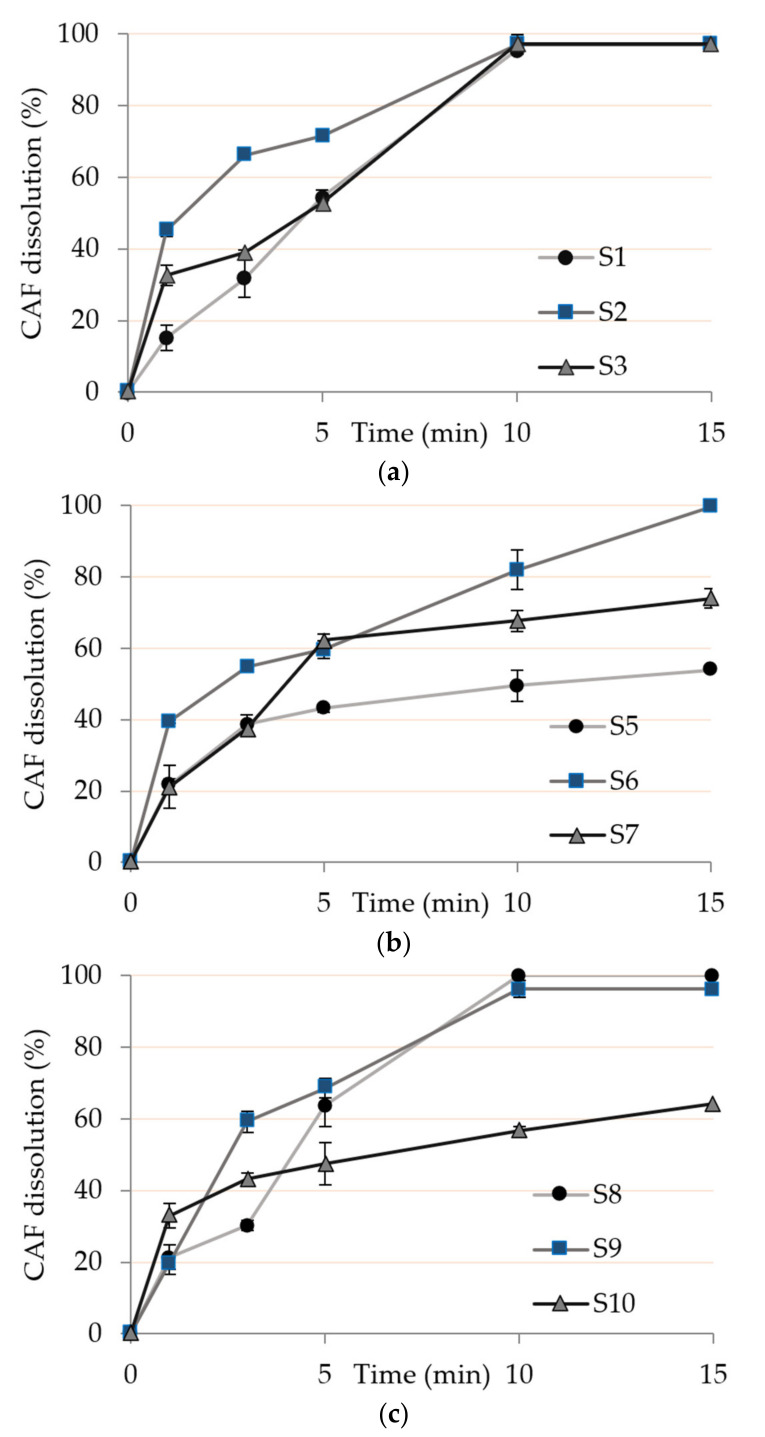
Dissolution profiles of caffeine (CAF) from the investigated samples: (**a**) subset I; (**b**) subset II and (**c**) subset III

The cumulative percentages of CAF released from different orodispersible films as a function of time are presented in Figure 4. There were no remarkable differences in dissolution profiles of the investigated samples during the first five minutes. Printed CAF was predominantly deposited on the surface of orodispersible thin films, thus drug dissolved almost immediately upon contact with dissolution media (Figure 4a). Interestingly, more than 80% of CAF was released within 10 min from all the subset I samples and subset III samples S8 and S9 indicating that, despite differences in structure, thickness and porosity, both sides open structure enables, also, fast drug release (Figure 4a,c). Sample S10, which had the highest thickness, exhibited slightly prolonged CAF release probably due to the multidimensional structure and longer drug diffusion distance. Drug release profiles were in rank-order with the determined film disintegration times. Although somewhat slower CAF dissolution was observed from the subset II, (Figure 4b), more than 80% CAF was released from all the investigated samples within 30 min. Standard deviation within triplicate samples was in the range from 0.35 to 6.07%. This implies rather low variability of data.

### 3.9. Printability Evaluation

**Figure 5:**
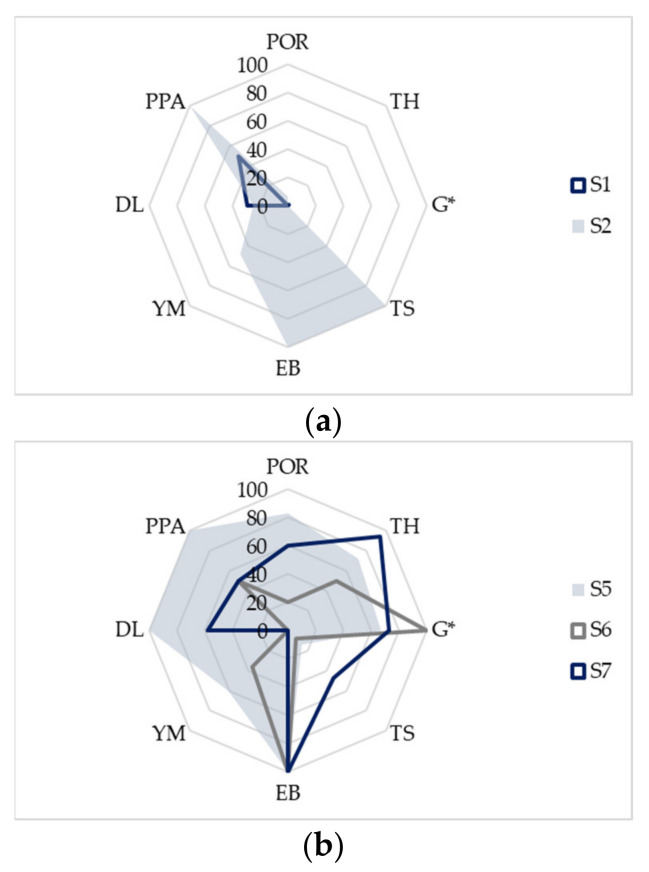

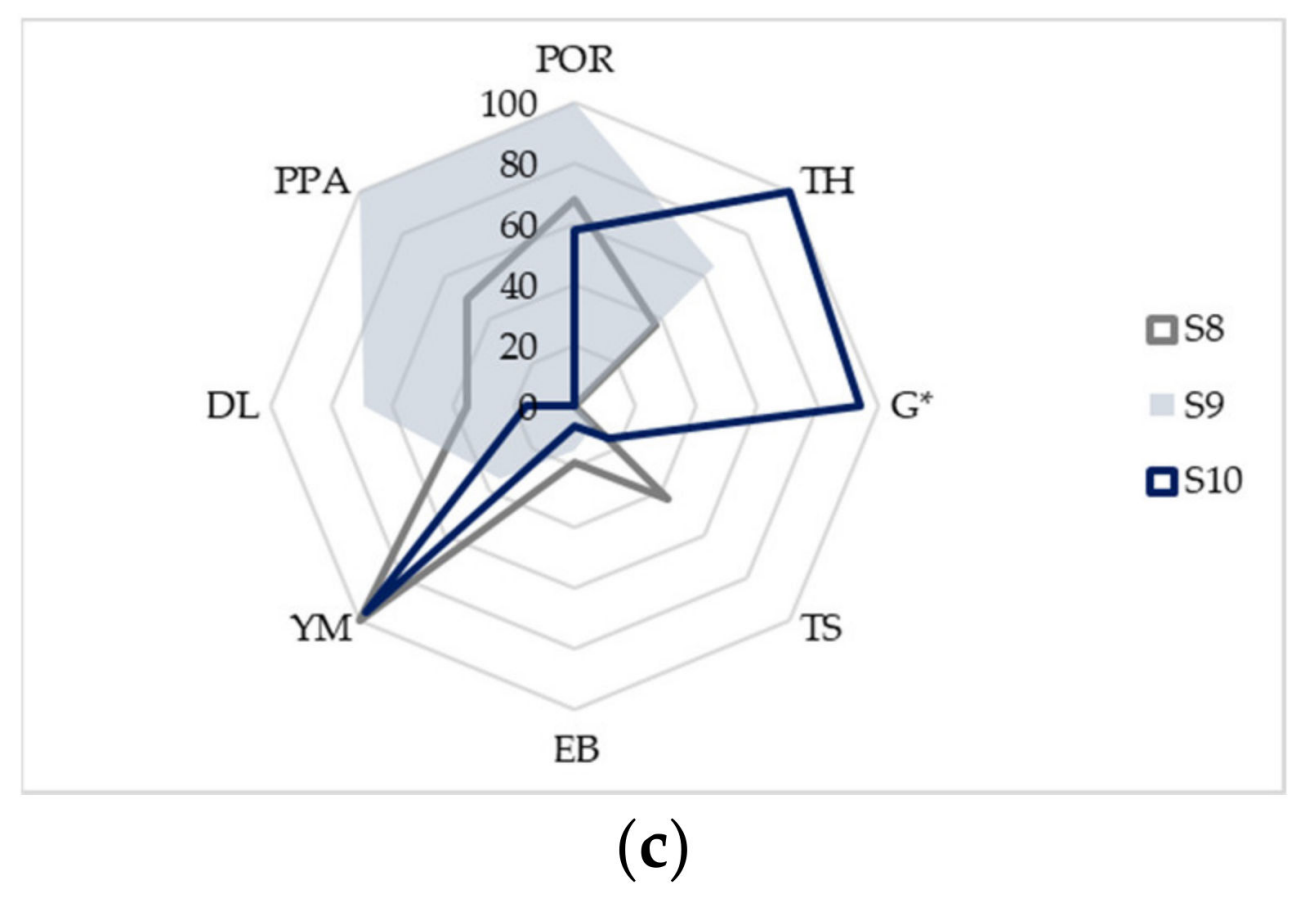
Radar charts for printability assessment of the investigated samples: Subset I (**a**); subset II (**b**) and subset III (**c**). POR—porosity, TH—thickness, DL—drug load and PPA—printed pattern appearance.

The comprehensive results of the investigated substrate printability evaluation are presented in Figure 5 as radar charts of the selected performance indicators. Experimentally determined parameters mathematically transformed, in order to standardize the values and facilitate printability comparison. The higher radar chart factor values are considered favorable (0%—not acceptable, 100%—acceptable) and the higher radar chart surface area indicates better printability. Boundaries suggested by Visser et al. for mechanical properties correspond to 100% [8]. The highest porosity, thickness and complex modulus values as well as the highest printed pattern score correspond to 100%. Scores from 0 to 100% were drawn on radar charts.

Sample S3 was eliminated from printability assessment, due to observed CAF crystallization and excessive brittleness, which was also reflected in the very high Young’s modulus value. Radar chart for the subset I samples (Figure 5a) revealed that sample S2 has higher relative surface area (20.4%) compared to sample S1 (6.9%). Mechanical properties were the main factor that contributed to sample S2 better printability, as these films were mechanically stronger but, at the same time, flexible enough to endure printing. The opposite, lower brittleness in conjunction with poor mechanical resistance observed in the sample S1 negatively affected its printability. The appearance of the printed patterns was also favorable in the case of S2 sample contributing to its overall performance as the drug printing substrate.

Within the subset II, sample S5 had notably higher relative surface area (59.2%) in comparison to samples S6 (26.1%) and S7 (42.1%). As the elongation at break was higher than 10%, its contribution was equal for all three samples. Tensile strength and Young’s modulus values were not critical for printability the of subset II samples, as they exhibited high flexibility, with appropriate tensile strength and Young’s modulus values. High drug load and porosity were main discriminative factors. Sample S5 had the highest values of those two parameters, as well as the favorable printed pattern appearance. It can be assumed that similarity in structure of samples S5 and S7 contributed to similarity in factors that affected their printability, while S6 was structurally different and complex modulus was the main parameter that positively affected its printability.

Within the subset III, samples S8 and S10 had similar relative surface areas (23.5% and 23.7%, respectively), although different parameters contributed to their printability. Sample S9 exhibited slightly higher relative surface area (29.9%) as higher porosity, and consequently higher drug load, with the good printed pattern appearance which positively affected its printability. Elongation at break values of the investigated wafer edible sheets were lower than 10%, so this parameter did not affect the subset III sample printability. The main difference between S8 and S10 samples printability was higher thickness and complex modulus for S10. As mentioned previously, high thickness values could indicate higher drug load, which was not the case with sample S10 as its drug load was not remarkable higher.

## 4. Conclusions

The results obtained indicate that different types of printing substrates may be used for drug inkjet printing. Although single polymer thin films with targeted mechanical properties could be used as printing substrates, their major disadvantage is low drug load, since drug deposition is limited to the film surface. Structured orodispersible film templates provided substantial advantages over the single polymer films with regards to the amount of drug incorporated. The appropriate combination of particulate matrix material and base polymer is important to ensure uniform porosity and good mechanical properties. Wafer edible sheets had comparable drug load to structured orodispersible film templates, but their mechanical properties were limiting for multiple printing cycles.

Construction of radar charts enabled visualization of relative contribution of each of the parameters evaluated on the investigated substrates printability and facilitated their comparative analysis. Differences in substrate structure governed which parameters predominantly affected their printability. Printability of single-polymer thin films was mainly dependent on the elongation at break and tensile strength values. Major challenge is to obtain good balance between flexibility and brittleness in order to avoid excessive stretching or tearing of thin films. In the case of structured orodispersible film templates it was evident that porosity was the key contributor to high drug load and more porous films had overall better printability characteristics. Furthermore, Young’s modulus and complex modulus must be taken into consideration as porous films can be overly rigid which negatively affects their printability due to possible rupture during the printing. Additionally, the printed pattern appearance could be useful indicator of substrate printability and included in printability evaluation. The obtained results provide new insight into the printing substrate characteristics and can possibly contribute to development of printability scoring system that could facilitate substate selection and production.

## Figures and Tables

**Table 1:** Sample composition.

Constituents (% *w/w*) *							
Subsets	Samples	HPC	PVA-PEG	SA	MDX	Purified Water (up to)	Absolute Ethanol (up to)
I	S1	7.0				100.0	
	S2		7.0			100.0	
	S3			7.0		100.0	
	S4				5.0	100.0	
II	S5	7.0	5.0				100.0
	S6	7.0		5.0			100.0
	S7	7.0			5.0		100.0
III	S8	Corn starch, olive oil, water					
	S9	Corn starch, olive oil, water					
	S10	Corn starch, olive oil, maltodextrin, water					

* 1% glycerol was added to samples S1–S7; HPC—hydroxypropyl cellulose, PVA-PEG—polyethylene glycol–polyvinyl alcohol graft copolymer, SA—sodium alginate, MDX—maltodextrine.

**Table 2:** Printing substrate characteristics.

Subsets	Sample	Weight (mg/cm^2^)			Thickness * (μm)			Drug Load * (μg)			Porosity *(Experimental) (%)			Porosity (Image Analysis) (%)	Printed Pattern Appearance (Color Uniformity/Edges Definition) ***	
**I**	S1	13.2	±	1.1	124	±	5	197.5	±	0.2	1.3	±	0.1	2.5	−	−
	S2	16.2	±	0.6	121	±	2	181.5	±	0.4	1.8	±	0.2	3.1	+	+
	S3	13.6	±	0.4	78	±	4	54.2	±	2.6	0.8	±	0.1	0.5	+	−
	S4	8.4	±	0.4	69	±	4	n/a **			0.8	±	0.1	0.3	n/a **	
II	S5	31.1	±	0.7	396	±	22	437.1	±	2.5	8.2	±	0.5	31.8	+	+
	S6	26.5	±	0.7	309	±	12	97.8	±	4.9	3.0	±	0.6	9.8	−	+
	S7	30.9	±	1.0	481	±	18	294.2	±	0.0	6.3	±	0.5	31.5	−	+
III	S8	8.6	±	0.0	264	±	6	217.3	±	4.1	6.9	±	0.2	33.8	−	+
	S9	12.4	±	0.2	369	±	6	333.2	±	1.0	9.7	±	0.2	41.1	+	+
	S10	18.6	±	0.2	502	±	7	151.0	±	5.6	6.1	±	0.4	30.4	+	+

* mean ± standard deviation; ** n/a—not applicable—sample disintegrated upon contact with ink during printing and *** + acceptable, − poor.

**Table 3:** Investigated samples disintegration before and after five printing cycles.

Disintegration Time							
Subsets	Samples	Before Printing *			After Printing *		
**I**	S1	27.0	±	2.0	26.7	±	1.0
	S2	32.5	±	0.8	2.2	±	1.8
	S3	41.7	±	0.9	41.5	±	2.0
	S4	3.5	±	0.5	n/a **		
**II**	S5	28.2	±	1.9	28.8	±	1.6
	S6	52.5	±	1.4	53.1	±	1.6
	S7	46.0	±	1.3	46.5	±	2.1
**III**	S8	17.2	±	1.2	17.0	±	0.9
	S9	20.8	±	0.7	20.5	±	1.0
	S10	129.3	±	2.5	129.3	±	1.4

* mean ± standard deviation and ** sample disintegrated upon contact with ink during printing.

**Table 4:** Mechanical properties of the investigated printing substrates

Subsets	Sample	EB * (%)			TS * (MPa)			YM * (MPa)			G* (MPa)		
**I**	S1	272.91	±	0.00	0.43	±	0.00	2.99	±	0.00	0.58	±	0.06
	S2	21.14	±	1.07	8.26	±	0.36	146.25	±	0.07	0.73	±	0.12
	S3	2.50	±	0.37	53.63	±	6.67	3498.00	±	173.09	90.35	±	4.34
**II**	S5	11.31	±	1.66	1.52	±	0.16	182.67	±	8.95	433.12	±	15.34
	S6	28.77	±	8.43	1.07	±	0.13	111.86	±	13.33	642.98	±	16.12
	S7	11.27	±	0.52	4.11	±	0.31	403.67	±	36.18	467.63	±	28.98
**III**	S8	1.86	±	0.21	3.81	±	0.66	300.45	±	0.64	0.35	±	0.06
	S9	1.49	±	0.31	1.14	±	0.53	105.49	±	16.84	2.65	±	0.07
	S10	0.67	±	0.18	1.66	±	0.38	290.20	±	22.77	601.29	±	0.08

* mean ± standard deviation. EB—elongation at break, TS—tensile strength, YM—Young’s modulus and G*—complex modulus.

## Data Availability

Not applicable.

## Supplementary Materials

The following are available online at https://www.mdpi.com/article/10.3390/pharmaceutics13040468/s1, Figure S1: Viscosity flow curve, Table S1: Characteristics of ethanol, liquid vehicle and ink.
