# Supplementary material for: An Investigation into Mechanical Properties and Printability of Potential Substrates for Inkjet Printing of Orodispersible Films
Source: Pharmaceutics. 2021 Mar 30;13(4):468. doi: 10.3390/pharmaceutics13040468 (PMC8066070; doi:10.3390/pharmaceutics13040468)
Supplement: Supplementary file 1 [file pharmaceutics-13-00468-s001.pdf]

pharmaceutics

Supplementary Materials: An Investigation into Mechanical Properties and Printability of Potential Substrates for Inkjet Printing of Orodispersible Films

Erna Turković, Ivana Vasiljević, Milica Drašković, Nataša Obradović, Dragana Vasiljević and Jelena Parojčić

Printing dispersion (ink) characterization

Liquid vehicle used for inkjet printing was ethanol:glycerol mixture (7:3). Then, 10 mg/mL caffeine anhydrous (CAF) was dissolved in the solvent mixture and hydrosoluble food dye was added to facilitate visualization of the printed patterns. The printing dispersion (ink) was characterized with respect to viscosity, density, surface tension and light refraction (comparative data on density, surface tension and refraction are also provided for the liquid vehicle without caffeine, and ethanol). Results are summarized in Table S1.

Rheological characteristics were evaluated using a rotary rheometer (Rheolab MC 120, PaarPhysica, Stuttgart, Germany) under the shear rates (D) from 1 to 200 s^{-1} with cylindric measuring device Z2 (radius 22.5 mm and length 67.5 mm) at 20 ± 0.2 °C. Values for the maximal (η_{max}) and minimal (η_{min}) apperent viscosity were obtained (at the low (6 s^{-1}) and high (200 s^{-1}) shear rates, respectively) from the viscosity flow curve (Figure S1). The measurements were performed in triplicate.

Relative density of liquids was determinated using Gay–Lussac pycnometer (according to the Ph.Eur. 10 (2.2.5)) [1]. Pycnometer was weighed empty, filled with purified water, and filled with the investigated liquids and the average of six measurements for was used in Equation (S1):

$$\varrho = ((m_{investigated\ liquid} - m_{pycnometer})/(m_{water})) \times \varrho_{water} \quad (S1)$$

where ϱ and m represent the density and mass, respectively.

Refractive index (n) of ethanol, etanol:glycerol mixture and ink was determined using a refractometer (ABBE bench refractometer, 2WAJ, OPTIKA microscopes, Ponteranica, Italy). The refractometer was set up to measure the refractive index at the wavelength corresponding to the D-line of sodium (589.3 nm). The average of six measurements was reported.

Surface tension was measured using stalagmometer (Krüss GmbH, Hamburg, Germany) by counting the falling drops every 5 s during 60 s. Average value for six measurements is presented.

Table S1. Characteristics of ethanol, liquid vehicle and ink.

Samples	Viscosity (mPa × s)	Density (g/mL)	Refractive index	Surface tension (nN/m)
Ethanol	n/a	0.79	1.36	22.69
Ethanol: glycerol (7:3) mixture	n/a	0.89	1.39	22.75
Ink	η_{max} (D* = 6.1 s^{-1}) = 48.4<br>η_{min} (D* = 200 s^{-1}) = 2.9	0.90	1.39	22.85

*D—shear rate, n/a—not applicable.

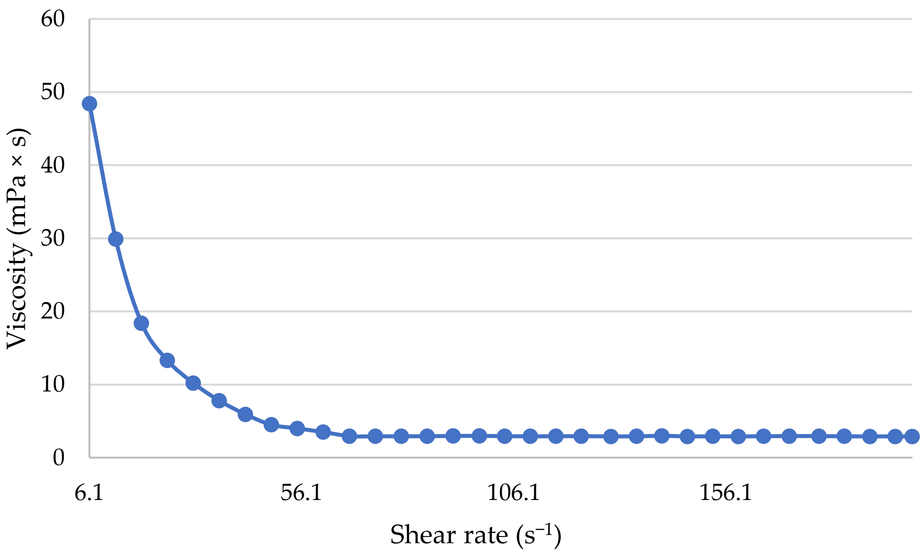

Figure S1. Viscosity flow curve.

References:

1. Council of Europe, Monograph 2.2.5: Relative density, 10th edition, European Pharmacopoeia, Strasbourg, 2019.
